# Influence of Hip Abductor Strength on Running Biomechanics in Healthy Populations: A Scoping Review

**DOI:** 10.1186/s40798-026-01009-w

**Published:** 2026-03-24

**Authors:** Sebastian Landauer, Andreas Konrad, Florian Kurt Paternoster

**Affiliations:** 1 Biomechanics in Sports, Technical University of Munich, Munich, Germany; 2 Institute of Human Movement Science, Sport and Health, University of Graz, Graz, Austria

**Keywords:** Gluteus medius, Hip external rotators, Hip strength, Running mechanics, Running kinematics, Running kinetics

## Abstract

Current evidence provides both rationale and preliminary support for an association between hip abductor weakness and detrimental running biomechanics. Previous reviews suggest a potential, but inconsistent, role of hip abductor strength in controlling lower limb biomechanics, with conclusions varying depending on the methodology, population, and task. This scoping review aimed to identify and map all available evidence about the influence of hip abductor strength on running biomechanics in healthy runners comparing study designs and methods. Four databases (PubMed, Web of Science, Scopus, and SPORTDiscus) were systematically screened for peer-reviewed articles in English investigating the influence of hip abductor strength on running biomechanics in healthy runners. Running biomechanics were defined as any quantitative kinematic (e.g., joint angles) or kinetic (e.g., joint moments) outcome. Eligible studies were synthesized and presented in a summary table, including general and experimental study characteristics and outcome results. After removing duplicate entries, 1322 records were identified, and 19 articles found to be eligible. These studies examined a wide range of gait cycle characteristics across a diverse set of biomechanical variables. Most studies focused on the hip (*n* = 16) followed by the knee (*n* = 8), and pelvis (*n* = 7). Fewer studies examined the trunk (*n* = 3), followed by the ankle (*n* = 2), and tibia (*n* = 1). Frequently reported outcomes included peak angles, range of motion, angular excursion, and peak velocity. Study designs and methodologies varied considerably. Most studies were cross-sectional (*n* = 17), while only two were intervention trials, both with notable methodological limitations. Overall, the evidence provides insufficient support for a consistent association between hip abductor or external rotator strength and running biomechanics in healthy runners, and no convincing evidence that strengthening these muscles alone leads to systematic or predictable changes in running kinematics or kinetics. These findings challenge commonly held assumptions regarding the biomechanical effects of hip strengthening and highlight the need for well-controlled intervention studies with greater methodological consistency. Future research should prioritize experimental designs that test hip strengthening under specific conditions, such as fatigue or higher running speeds, to determine whether meaningful biomechanical effects occur.

## Key Points

- Seventeen of the 19 included studies were cross-sectional, and collectively, the evidence provides little consistent support for a link between hip abductor or external rotator strength and running biomechanics in healthy runners.
- Only two intervention studies have tested whether targeted hip strengthening can alter running biomechanics, and their conflicting results offer no clear evidence of systematic changes.
- Future research should focus on well-controlled intervention studies with greater methodological consistency, particularly under conditions such as fatigue or higher running speeds, to clarify whether hip strengthening can meaningfully influence running biomechanics.

## Background

As one of the most widely practiced activities around the world [1], running has gained recognition as a key lifestyle intervention for longevity and health [2–4]. At the same time, running carries a considerable risk of injury, with a mean incidence of 37% [5] and njury rates ranging from 2.5 injuries per 1000 h of running in track and field athletes up to 33.0 among novice runners [6]. A previous running-related injury is among the strongest risk factors for sustaining future running-related injuries [7–9]. While injury history is unchangeable, biomechanical factors offer a modifiable pathway to reducing injury risk [10]. Hip adduction, for example, has been linked to injury, particularly in female runners [11–13].

Powers et al. (2010) proposed that weak hip abductors may contribute to excessive hip adduction, a movement pattern commonly observed in injured runners [14]. Consistent with this hypothesis, cross-sectional studies have associated reduced hip abductor strength (HABDS) with iliotibial band syndrome [15]. Gluteal muscle weakness may also be linked to a broader cascade of altered lower-limb movements, including pelvic drop, increased femoral internal rotation, knee valgus stress, patellar displacement, and tibial external rotation [16]. These movement alterations have been associated with overuse injuries in runners [17].

At the knee, the most common site of running-related injury [5, 17], hip abductor weakness may influence joint loading through two distinct mechanisms. First, a contralateral pelvic drop (Trendelenburg sign) shifts the body’s center of mass away from the stance limb, increasing the knee varus moment. Second, a compensatory contralateral trunk lean (compensated Trendelenburg sign) can increase the knee valgus moment [14, 17].

In addition to hip abductor capacity, hip external rotator strength (HERS) may also be relevant because the gluteus medius, the primary hip abductor, has two functional roles. While it stabilizes the pelvis in the frontal plane, its posterior portion also contributes to hip external rotation [18]. As a result, insufficient external rotator capacity may allow greater femoral internal rotation during stance, potentially magnifying the effects of hip abductor weakness on dynamic knee valgus and knee joint loading during running. This rationale is supported by a preliminary literature search which identified several studies that have examined both HABDS and HERS in relation to running biomechanics [19–21].

Taken together, current evidence provides both rationale and preliminary support for an association between hip abductor or external rotator weakness, altered biomechanics, and injury. However, it remains unclear whether these factors precede injury or emerge as compensatory responses [22]. Retrospective comparisons between injured and uninjured runners cannot distinguish between cause and effect [23]. To draw a clear connection between weak hip abductors or external rotators, detrimental running biomechanics, and injury, we must understand the relationship between HABDS or HERS and running biomechanics before injury occurs.

The influence of HABDS or HERS on biomechanics has been the subject of several reviews, yet their findings remain inconsistent [16, 24–29]. Some authors highlighted the crucial role of the gluteal muscles in maintaining frontal plane knee position across different movements [16], while others found at least moderate support for hip strengthening interventions to alter lower extremity kinematics [29]. In contrast, further reviews found only limited or inconsistent associations between hip abductor weakness and knee valgus [24, 27] or hip adduction and knee internal rotation [28]. Moreover, one review concluded that frontal plane knee mechanics could be improved when hip strengthening was combined with neuromuscular control exercises in females [26]. Adding to this complexity, a meta-analysis reported that hip strength was linked to increased knee valgus exclusively during single-leg ballistic landings, but not in double-leg or non-ballistic squat tasks [25]. The relationship between HABDS or HERS and biomechanics may only reveal itself with more demanding tasks that sufficiently challenge lower limb biomechanics, such as single-leg jumping or running [25].

In sum, these reviews suggest a potential, but inconsistent, role of HABDS or HERS in controlling lower limb biomechanics, with conclusions varying depending on the methodology, population, and type of movement task under investigation. One explanation for these inconsistencies may lie in the lack of task specificity. In all except one case [28], reviews pooled evidence across diverse movements, making it difficult to directly transfer findings to running. Since running can be considered a sequence of ballistic single-leg landings, task-specific analyses may be crucial for identifying relevant associations [25]. However, the only review that focused exclusively on running biomechanics considered general strength training rather than isolating HABDS or HERS [28]. This leaves a clear research gap. The specific relationship between HABDS or HERS and running biomechanics remains to be determined.

Based on the PCC (population, concept, and context) elements, our research question was: What evidence exists about the influence of HABDS or HERS on running biomechanics in healthy runners? We raised this question to identify and map every source independently of the methodological approach and to provide an overview of the biomechanical variables that were investigated [30]. Our secondary research question was: What study designs and methods were used by researchers in this area? We aimed to compare them and provide recommendations where more primary research is needed and how it should be conducted.

## Methods

### Protocol and Registration

Our protocol followed the PRISMA Extension for Scoping Reviews [31] and the JBI Manual for Evidence Synthesis regarding Scoping Reviews [32] and was registered prospectively with the Open Science Framework (10.17605/OSF.IO/5VBNG) on November 20, 2023. This review was conducted as a scoping review because the existing literature on HABDS and running biomechanics is highly heterogeneous, spanning a wide range of biomechanical outcomes and study designs. A scoping approach was therefore considered appropriate to map the available evidence, describe how studies have been conducted, and identify gaps in the literature, rather than to address a narrowly defined question suitable for systematic review or meta-analysis.

### Eligibility Criteria

We included cross-sectional and longitudinal prospective studies that investigated the influence of HABDS or HERS on running biomechanics. The target population was not restricted based on general characteristics such as age or body mass index (BMI). However, only studies with healthy runners without acute running-related pain or injury were included. No predefined minimum duration for being pain- or injury-free was set. We defined running biomechanics as any quantitative kinematic (e.g., joint angles) or kinetic (e.g., joint moments) outcome derived from motion capture and its combination with force plates for inverse dynamics. We only included peer-reviewed journal papers if they were available in English, but excluded review articles, conference proceedings, book chapters, theses or dissertations, and other non-peer-reviewed materials such as reports or editorials. We also excluded studies focusing on walking, sprinting, or other non-steady-state running tasks, studies investigating participants with clinical conditions or ongoing pain, and studies reporting only electromyography outcomes without kinematic or kinetic data. If intervention studies combined hip abductor or external rotator strengthening with other components (e.g. gait retraining), we included them but analyzed them separately from studies using strengthening alone.

### Information Sources

We searched PubMed, Web of Science, Scopus, and SPORTDiscus and customized our search to each database. Finally, we supplemented the search results by scanning reference lists of all included articles.

### Search

In consultation with a university librarian, we developed a search strategy with three main concepts: HABDS, running, and biomechanics. We iteratively collected synonyms through provisional searches and their respective results and combined all synonyms within each concept with the Boolean operator OR. We divided HABDS into two sub-concepts, hip and strength, and connected them via proximity operators in all search platforms except PubMed. Since proximity searching is unavailable in PubMed, we connected the two sub-concepts, like all other concepts, with the Boolean operator AND. We first developed the search algorithm in PubMed and translated it to other platforms (Electronic Supplementary Material Appendix 1). The search strategy and its translations in all search platforms are available online at the Open Science Framework mentioned above. We conducted the final search on November 20, 2023, and updated it on April 30, 2024. We exported the search results to Citavi 6 (Swiss Academic Software GmbH) and removed all duplicates.

### Selection of Sources of Evidence

Two members (SL and FP) of our team independently screened the results for inclusion of relevant articles. We preselected articles according to their title and abstract and screened them as full texts for final decision. We consulted a third reviewer (AK) in case of disagreement. To increase interrater reliability, we initially screened the first 50 articles, discussed our results, and refined the inclusion and exclusion protocol accordingly. This led to an inter-rater reliability of 99.62% with a Kappa coefficient of 0.88 in the final screening process.

### Data Charting Process

We (SL and FP) developed a data-charting form in Microsoft Excel to compile general study characteristics (i.e. year of publication and author names), experimental characteristics (i.e. sample size, sex, subject characteristics [age, height, body mass], running level, running volume, running speed, running surface, independent variables [HABDS/HERS] and dependent variables [biomechanical variables], statistical measures) and outcome results. We pilot-tested the charting form using three included articles with different designs and revised it accordingly.

## Results

### Selection of Sources of Evidence

**Fig. 1:**
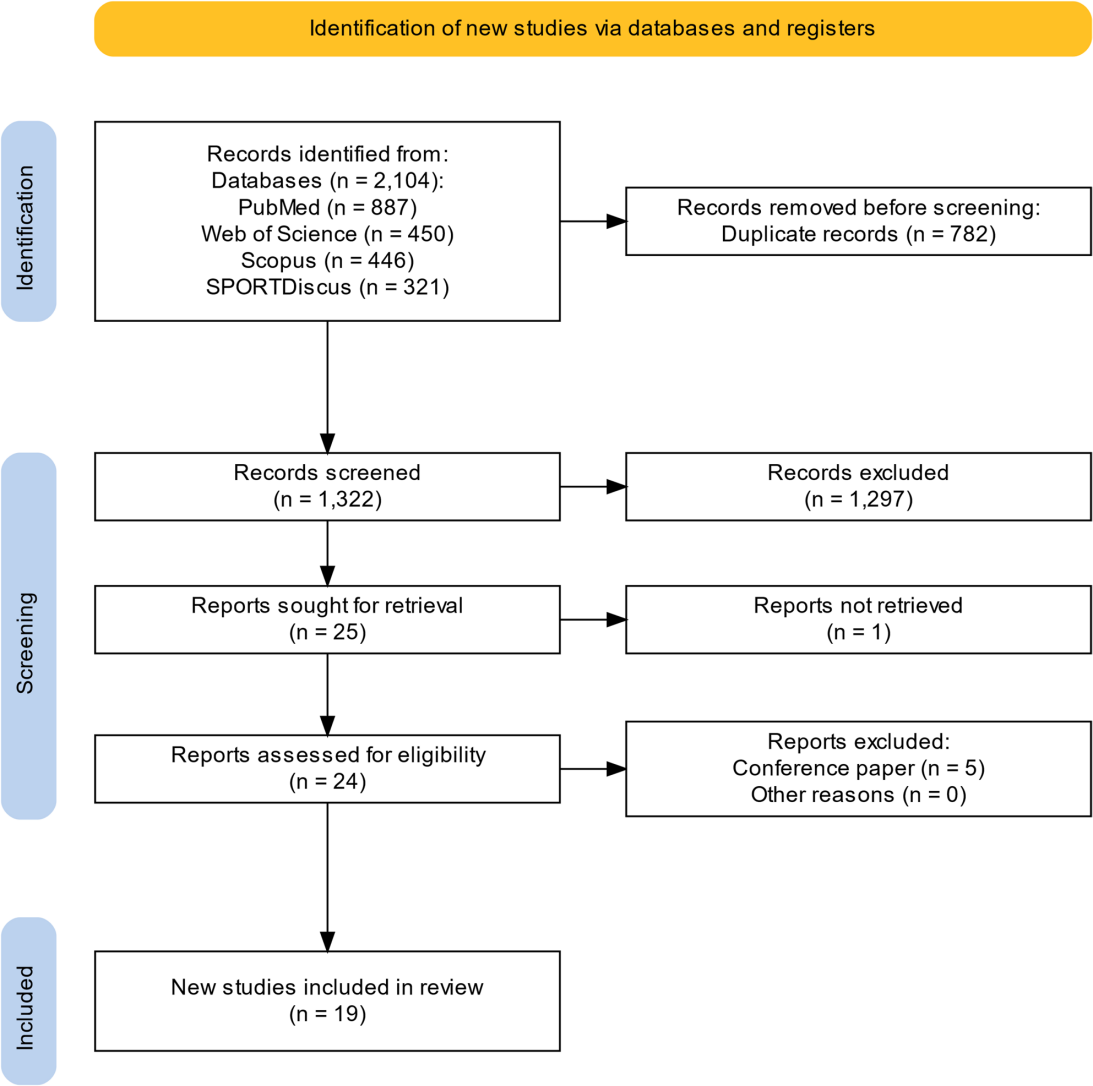
PRISMA study identification and selection flow (developed with the Shiny app for producing PRISMA 2020 [33])

Figure 1 displays a PRISMA flow chart [33] of the selection process. After the removal of duplicates, we screened 1322 records by title and abstract with 25 reports eligible for full text retrieval. One article was not available as full text [34] and five records were conference abstracts [35–39]. The remaining 19 studies were included in this scoping review. No further records were found by scanning the reference lists of the included studies.

### General Characteristics

All the eligible 19 studies were published between 2008 and 2022. They included a total sample size of 683, with 372 females and 311 males. Nine studies included females [20, 21, 40–46], three included males [47–49], and seven studies had mixed-sex samples [19, 50–55]. Participants’ mean age ranged from approximately 18 to 38 years, with the exception of one study that included 35 older runners with a mean age of approximately 60 years [53]. Participants in all studies had a BMI within the normal weight range according to WHO standards for classification [56] except one study group that had a BMI over 25 kg/m² [44] and another with a BMI close to 25 kg/m² [43].

Fifteen studies included recreational runners with a broad range of training volumes, from general physical activity that involved some running [40, 43, 44, 48] to dedicated running training with average weekly mileages of up to 76 km [19, 20, 41, 42, 46, 47, 50, 51, 54]. One study reported the training volume in hours per week [53], while another did not report the training volume at all [21]. However, both described their samples as recreational runners. In contrast, only four studies included collegiate cross-country runners with mileages over 20 km/week [49], over 40 km/week [45], and 103 ± 29 km/week [52]. The fourth study described their study population as cross-country runners but with no explicit measure of running volume [55].

### Outcome Results

**Fig. 2:**
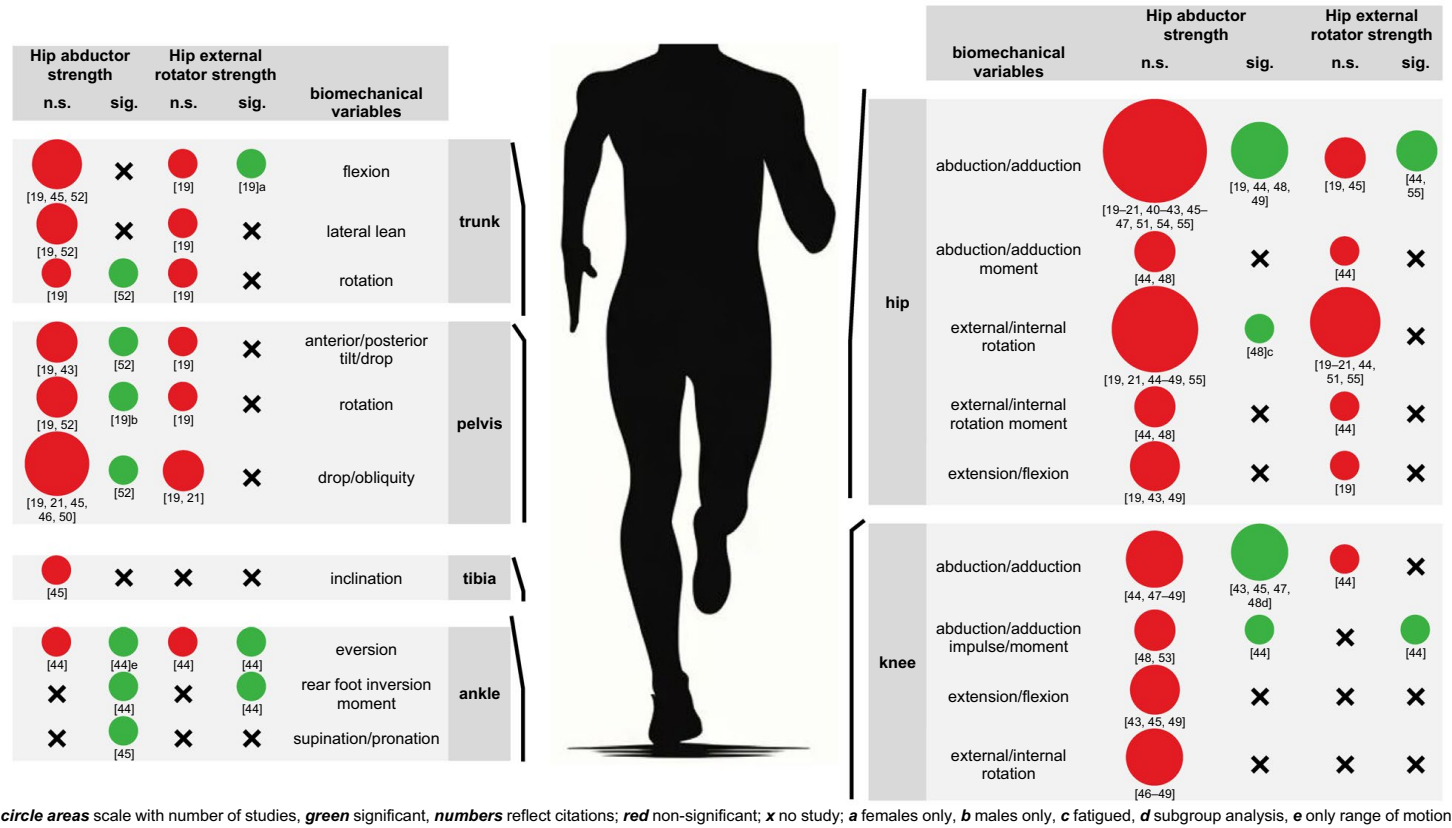
Significant (sig.) or non-significant (n.s.) associations between hip abductor or hip external rotator strength and biomechanical variables. circle areas scale with number of studies, green significant, numbers reflect citations; red non-significant; x no study; a females only, b males only, c fatigued, d subgroup analysis, e only range of motion

Figure 2 maps the available evidence independently of the methodological approach and gives an overview of the running biomechanical variables examined by different authors. It visualizes the number of studies that reported significant or non-significant associations with HABDS or HERS. The variables are in order from proximal to distal, and their names cover the terminology derived from all studies. Table 1 shows the characteristics and results of all included studies.

Sixteen studies investigated the influence of HABDS or HERS on the biomechanics of the hip [19–21, 40–49, 51, 54, 55]. The knee was examined in eight studies [43–49, 53] and the pelvis in seven studies [19, 21, 43, 45, 46, 50, 52]. Less attention was paid to the trunk with three studies [19, 45, 52], the ankle with two studies [44, 45], and the tibia with one study [45].

#### Trunk

Three studies investigated trunk movements including flexion [19, 45, 52], lateral lean [19, 52], and rotation [19, 52]. While most combinations of HABDS and HERS with trunk biomechanical variables were non-significant, two studies observed significant associations. Specifically, greater HABDS was associated with reduced trunk rotation (*r* = -0.53 to -0.57; *p* < 0.05) [52] and greater HERS was associated with reduced trunk flexion excursion in females (*r* = -0.41; *p* < 0.05 or *p* < 0.0056 with alpha-level correction), but not in males [19].

#### Pelvis

Seven studies examined pelvic movements covering anterior/posterior tilt or drop [19, 43, 52], rotation [19, 52], and lateral drop or obliquity [19, 21, 45, 46, 50, 52]. Most outcomes were non-significant and the significant findings again arose from individual studies. Greater HABDS was associated with reduced pelvic tilt (*r* = -0.32 to -0.35; *p* < 0.05) [52], reduced pelvic internal rotation excursion in males (*r* = -0.33; *p* < 0.05 or *p* < 0.0056 with alpha-level correction) [19], and reduced pelvic obliquity (*r* = -0.44 to -0.50; *p* < 0.05) [52].

#### Hip

Sixteen studies investigated hip movements, including abduction/adduction [19–21, 40–49, 51, 54, 55], external/internal rotation [19–21, 44–49, 51, 55], and extension/flexion [19, 43, 49]. Two studies additionally considered joint moments related to abduction/adduction and external/internal rotation but with no significant result [44, 48]. Most kinematic outcomes were non-significant, but significant correlations were reported for abduction/adduction [19, 44, 48, 49, 55] and external/internal rotation [48]. Specifically, greater HABDS was associated with reduced maximum hip adduction velocity (*r* = -0.32; *p* = 0.049) [48], reduced hip adduction excursion in females (*r* = -0.41; *p* < 0.05 or *p* < 0.0056 with alpha-level correction) [19], but not in males, and reduced hip abduction/adduction range of motion (*r* = -0.46; *p* = 0.008) [49]. In contrast, hip adduction range of motion increased following a hip abduction and hip external rotation strengthening program (effect size [ES] = 0.33; *p* = 0.05) [44]. Greater HABDS was also associated with reduced hip internal rotation velocity (*r* = -0.41; *p* = 0.01), but only after a fatiguing run [48]. Finally, greater HERS was associated with reduced peak hip adduction (*r* = -0.29; *p* = 0.006) [55].

#### Knee

Eight studies examined knee movements, including abduction/adduction (varus/valgus) [43–45, 47–49], extension/flexion [43, 45, 49], and external/internal rotation [46–49]. Three studies additionally analyzed abduction/adduction impulse or moment [44, 48, 53]. Again, most outcomes were non-significant, but several significant associations were reported under specific conditions. Greater HABDS was associated with greater knee abduction/adduction range of motion at foot strike (*r* = 0.50; *p* < 0.05) and stance (*r* = 0.55; *p* < 0.05), but in the right limb only [45], as well as with greater maximum knee adduction (*r* = 0.39; *p* = 0.017) and greater maximum knee adduction velocity (*r* = 0.38; *p* = 0.019), but only after a fatiguing run [48]. A subgroup analysis showed that runners who simultaneously increased hip adduction and knee abduction between initial foot strike and 50% of stance, demonstrated, that an increase in eccenric HABDS of 1 Nm per kilogram of body mass corresponded to 2.8° greater knee adduction (*p* = 0.002) [47]. Post hoc comparisons between a weak and a strong HABDS group indicated a difference in knee abduction of approximately + 4° for the weak group (*p* < 0.05) [43]. Finally, knee abduction moment was significantly reduced (ES = 0.48; *p* = 0.05) after a hip abduction and hip external rotation strengthening program [44].

#### Tibia

There was no significant correlation between HABDS and tibial inclination angle at foot strike [45].

#### Ankle

Two studies investigated ankle movements, including eversion [44], rear foot inversion moment [44], and supination/pronation [45]. Significant reductions in eversion range of motion (ES = 0.53; *p* = 0.05) and rear foot inversion moment (ES = 0.83; *p* = 0.01) were observed after a hip abduction and hip external rotation strengthening program [44]. In addition, greater HABDS was also associated with greater supination at the left foot strike (bilateral: *r* = 0.48; right: *r* = 0.51; *p* < 0.05) and reduced peak pronation at the right stance (*r* = -0.54; *p* < 0.05) [45].

**Table 1:** General and experimental study characteristics with outcome results

Study	Study population: sample size | sex age height mass running level running volume			Pace surface	HABDS | HERS: movement (position) contraction mode tested side measurement device force | torque normalization	Study design (statistics)	Associations between HABDS or HERS and biomechanical variables
Baggaley et al. 2015 [40]	25 f 29 ± 6 years 1.67 ± 0.05 m 61.6 ± 11.3 kg recreational > 30 min 3 times/week			2.7 m/s (prescribed) treadmill	HABDS (side-lying) isometric side N/A stationary dynamometer torque (based on femur length) normalized to body mass	Cross-sectional (correlation)	*HABDS*: peak hip adduction (n.s.) hip adduction excursion (n.s.)
Brindle et al. 2020 [41]	*Small peak hip adduction*: 20 f 27 ± 7 years 1.66 ± 0.06 m 60.4 ± 6.6 kg recreational 37 ± 19 km/week		*Large peak hip adduction*: 20 f 27 ± 6 years 1.66 ± 0.05 m 65.5 ± 7.5 kg recreational 32 ± 13 km/week	3.7 (± 5%) m/s (prescribed) over ground	HABDS (side-lying) eccentric right hand-held dynamometer (manual fixation) force multiplied by moment arm normalized to body mass and height	Cross-sectional (t-tests)	*Eccentric HABDS*: peak hip adduction (n.s.) (no group difference between large and small peak hip adduction group; 6.7° larger in large group*)
Brund et al. 2018 [47]	100 m 38 (IQR: 21) years 1.82 (IQR: 0.08) m 79 (IQR: 12) kg recreational 25 (IQR: 14) km/week			2.78 m/s (prescribed) treadmill	HABDS (posture N/A) eccentric right and left (combined) isokinetic dynamometer (30°/s; RoM: 0–20°) torque normalized to body mass	Cross-sectional (regression)	*Eccentric HABDS*: knee abduction (n.s) knee internal rotation (n.s.) hip internal rotation (n.s.) hip adduction (n.s.) *knee valgus subgroup analysis*: 1 Nm/BW ↑ eccentric HABDS corresponds to 2.8° ↓ knee abduction (*)
Burnet and Pidcoe 2009 [50]	12 f | 9 m 25.2 ± 3.8 years 1.73 ± 1.0 m 70.6 ± 12.3 kg recreational 33.3 ± 18.7 km/week			2.98 ± 0.29 m/s (self-selected) treadmill	HABDS (side-lying) isometric right and left (separately) hand-held dynamometer (strap fixation) force multiplied by thigh length normalized to body mass	Cross-sectional (correlation)	*HABDS*: pelvic drop (n.s.) rate of change of pelvic drop (n.s.) (for both sexes together and individually and for every 2 min from 0 to 30 min)
Dierks et al. 2008 [51]	15 f | 5 m 22.7 ± 5.6 years 1.70 ± 0.08 m 63.02 ± 9.15 kg recreational 24.6 ± 10.3 km/week			2.64 ± 0.33 m/s (self-selected) treadmill	HABDS (side-lying) HERS (seated; 90° knee and hip flexion) isometric right or left (randomly) hand-held dynamometer (strap fixation) force multiplied by thigh (HABDS) or shank length (HERS) normalized to body mass	Cross-sectional (correlation)	*HABDS*: hip adduction at beginning (n.s.) hip adduction after 45.0 ± 12.0 min (n.s.) *HERS*: peak hip internal rotation at beginning (n.s.) peak hip internal rotation after 45.0 ± 12.0 min (n.s.)
Foch et al. 2020 [42]	25 f 25 ± 8 years 1.66 ± 0.07 m 59.3 ± 7.2 kg recreational and competitive 44 ± 23 km/week			2.8 ± 0.4 m/s (self-selected) treadmill	HABDS (side-lying) isometric side N/A stationary dynamometer torque normalized to body mass and height	Cross-sectional (correlation)	*HABDS*: hip adduction at foot contact (n.s.) peak hip abduction (n.s.) peak hip adduction velocity (n.s.) hip abduction excursion (n.s.)
Ford et al. 2013 [52]	10 f | 14 m 19.5 ± 1.5 | 20.2 ± 1.2 years 1.54 ± 0.31 | 177.8 ± 6.7 m 53.7 ± 4.3 | 64.0 ± 4.2 kg collegiate cross-country 103 ± 29 km/week			3.58 ± 0.26 m/s (self-selected) 3.58 m/s (prescribed) treadmill	HABDS (standing) concentric side N/A isokinetic dynamometer (120 °/s) torque normalized to body mass	Cross-sectional (correlation)	*HABDS (prescribed | self-selected speed)*: pelvic tilt: *r* = -0.35* | *r* = -0.32* pelvic obliquity: *r* = -0.50* | *r* = -0.44* pelvic rotation (n.s) trunk flexion (n.s) trunk lateral lean (n.s) trunk rotation: *r* = -0.57* | *r* = -0.53*
Fukuchi et al. 2014 [53]	*Younger:* 14 f | 21 m 28.9 ± 4.7 years 1.72 ± 0.09 m 67.9 ± 11.5 kg recreational 3.5 ± 1.9 h/week		*Older*: 13 f | 22 m 60.2 ± 4.2 years 1.71 ± 0.10 m 68.4 ± 11.0 kg recreational 3.2 ± 0.8 h/week	2.7 m/s (prescribed) instrumented treadmill	HABDS (side-lying) HERS (seated; 90° hip and knee flexion) isometric right hand-held dynamometer (strap fixation) force normalized to body mass	Cross-sectional (correlation)	*HABDS*: knee abduction impulse (n.s.) other variables: N/A
Hannigan et al. 2018 [19]	23 f | 37 m 27.4 ± 10.0 | 29.9 ± 10.7 years 1.60 ± 0.21 | 1.80 ± 0.08 m 56.2 ± 6.2 | 72.8 ± 12.2 kg recreational 72 ± 19 | 76 ± 33 km/week			3.3 ± 0.3 m/s (f) 3.5 ± 0.4 m/s (m) (self-selected) over ground	HABDS (standing; 10° hip abduction) HERS (seated; 90° hip and knee flexion) isometric right and left (combined) stationary dynamometer torque normalized to body mass	Cross-sectional (correlation)	*HABDS (f)*: hip adduction excursion: *r* = − 0.405* *HABDS (m)*: pelvic internal rotation excursion: *r* = − 0.330* *HERS (f)*: trunk flexion excursion: *r* = − 0.411* all other variables (n.s.)
Heinert et al. 2008 [43]	*Weakest HABDS*: 15 f (of 110 f) 23.4 ± 2.8 years 1.66 ± 0.08 m 69.0 ± 11.8 kg recreational > 3 times/week (aerobic)	*Strongest HABDS*: 15 f (of 110 f) 25.8 ± 6.7 years 1.63 ± 0.07 m 59.2 ± 6.1 kg recreational > 3 times/week (aerobic)		2.5 ± 0.7 m/s (weakest HABDS) 2.7 ± 0.6 m/s (strongest HABDS) (self-selected) treadmill	HABDS (side lying; 20° hip abduction) isometric dominant side hand-held dynamometer (anchoring station) force normalized to body mass	Cross-sectional (ANOVA)	*Weakest vs. strongest HABDS*: knee flexion (n.s.) hip flexion (n.s.) hip abduction (n.s.) pelvic tilt (n.s.) knee abduction* *post hoc comparisons*: +4° knee abduction at initial contact (weakest HABDS)
Liu et al. 2021 [54]	15 f | 14 m 25.2 ± 3.4 | 26.2 ± 4.5 years 1.7 ± 0.1 | 1.8 ± 0.1 m 59.2 ± 8.5 | 76.7 ± 8.6 kg recreational 25.3 ± 9.2 | 36.3 ± 30.7 km/week			4.0 m/s (prescribed) over ground	HABDS (side-lying) isometric dominant side stationary dynamometer torque normalized to body mass	Cross-sectional (regression)	*HABDS as a predictor of*: peak hip adduction (late swing) (n.s.) peak hip adduction (stance) (n.s.)
Radzak and Stickley 2020 [48]	38 m 21.6 ± 4.0 years 1.78 ± 0.08 m 76.0 ± 12.4 kg Army Training Corps volume: N/A			4.0 m/s ± 10% (prescribed) over ground	HABDS (side-lying) isometric right hand-held dynamometer (strap fixation) force multiplied by moment arm normalized to body mass	Cross-sectional (correlation)	*HABDS (rested) vs.*: maximum hip adduction velocity: *r* = -0.322* all other variables (n.s.) *HABDS (fatigued; graded exercise test* *ad 80% VO2max run to exhaustion) vs.*: maximum hip adduction velocity: *r* = -0.393* hip internal rotation velocity: *r* = -0.410* maximum knee adduction: *r* = 0.385* maximum knee adduction velocity: *r* = -0.378* all other variables (n.s.) *change in HABDS vs.*: change in any biomechanical variable (n.s.)
Rodriguez et al. 2020 [55]	28 f | 18 m 18-20 years 1.72 ± 0.11 m 61.0 ± 10.0 kg cross-country runners volume: N/A			N/A (± % of mean training pace) over ground	HABDS (supine) HERS (prone and seated) isometric right and left (separately) hand-held dynamometer (manual fixation) force multiplied by limb length normalized to body mass	Cross-sectional (correlation)	*HABDS*: peak hip adduction (n.s.) peak hip internal rotation (n.s.) *HERS (prone)*: peak hip adduction: *r* = 0.286* peak hip internal rotation (n.s.)
Schmitz et al. 2014 [20]	*Experienced*: 19 f 23 ± 3 years 1.66 ± 0.06 m 59.0 ± 6.5 kg recreational 32.5 km/week (mean)	*Novice*: 19 f 24 ± 3 years 1.66 ± 0.05 m 60.5 ± 7.6 kg recreational no running > 5 years		3.35 m/s (prescribed) treadmill	HABDS (side-lying) HERS (seated) isometric right hand-held dynamometer (strap fixation) force multiplied by femur length normalized to body mass	Cross-sectional (correlation)	*HABDS*: hip adduction angle (n.s.) *HER*: hip rotation angle (n.s.)
Snyder et al. 2009 [44]	15 f 21.9 ± 1.2 years 1.54 ± 0.05 m 63.6 ± 6.4 kg recreational 30 min/day (moderate)			3.5–4.5 m/s (prescribed) over ground	HABDS (side-lying) HERS (seated; 90° hip and knee flexion) isometric trained and untrained side (separately) hand-held dynamometer (strap fixation) force normalized to body mass	Longitudinal intervention (ANOVA)	*Strength (trained* | *untrained side)*: HABDS ↑* 12. % (ES = 0.68) | (n.s.) HERS ↑* 231% (ES = 1.0) | (n.s.) *kinematics*: eversion RoM ↓* (ES = 0.53) eversion at heel strike (n.s.) eversion velocity (n.s.) knee abduction RoM (n.s.) hip adduction RoM ↑* (ES = 0.33) hip internal rotation RoM (n.s.) *kinetics*: rear foot inversion moment ↓* (ES = 0.83) knee abduction moment ↓* (ES = 0.48) hip abduction moment (n.s.) hip external rotation moment (n.s.)
Taylor-Haas et al. 2014 [49]	33 m 18.3 ± 1.9 years 1.77 ± 0.06 m 61.6 ± 5.0 kg collegiate cross-country > 20 km/week			3.8 m/s (mean) (self-selected) treadmill	HABDS (standing) concentric right and left (combined) isokinetic dynamometer (120 °/s; full RoM: ca. 0–45°) torque normalized to body mass	Cross-sectional (correlation)	*HABDS*: hip abduction/adduction RoM: *r* = -0.462* hip flexion/extension RoM (n.s.) hip internal/external rotation RoM (n.s.) knee abduction/adduction RoM (n.s.) knee flexion/extension RoM (n.s.) knee internal/external rotation RoM (n.s.)
Venable et al. 2022 [45]	20 f 19.2 ± 1.1 years 1.6 ± 0.6 m 57.3 ± 5.2 kg intercollegiate cross-country > 40 km/week			N/A (self-selected) treadmill	HABDS (side-lying) concentric right and left (separately) isokinetic dynamometer (90°/s; 0–30° hip abduction RoM) torque normalized to body mass	Cross-sectional (correlation)	*HABDS*: right knee adduction at foot strike: *r* = 0.50* right knee adduction at stance: *r* = 0.55* left supination at foot strike: *r* = 0.48* right peak pronation during stance: *r* = -0.47* *right HABDS*: left supination at foot strike: *r* = 0.51* *left HABDS*: right peak pronation during stance: *r* = -0.54* all other combinations (n.s.)
Willy and Davis 2011 [46]	*Training* 10 f 22.7 ± 3.5 years N/A N/A (BMI: 22.3 ± 2.3 kg/m²) recreational 21.7 ± 8.5 km/week	*Control* 10 f age: 21.2 ± 2.2 years N/A N/A (BMI: 22.2 ± 2.9 kg/m²) recreational 22.5 ± 11.7 km/week		3.35 m/s (prescribed) treadmill	HABDS (side-lying) HERS (prone with neutral hip) isometric side N/A hand-held dynamometer (strap fixation) force multiplied by moment arm normalized to body mass	Longitudinal intervention (ANOVA)	*Strength (training group)*: HABDS ↑ 41. %* (ES = 1.39) HERS ↑ 241%* (ES = 0.62) *strength (control group)*: HABDS (n.s.) HERS (n.s.) *kinematics*: peak hip adduction (n.s.) peak hip internal rotation (n.s.) peak contralateral pelvic drop (n.s.) peak knee external rotation (n.s.)
	Both: peak hip adduction angle > 20° during running						
Zeitoune et al. 2020 [21]	29 f 29.9 ± 4.0 years 1.64 ± 0.03 m 60.6 ± 6.3 kg recreational N/A			2.92 m/s (prescribed) treadmill	HABDS (side-lying) HERS (prone; 90° knee flexion) isometric dominant / right side hand-held dynamometer (fixation N/A) force no normalization	Cross-sectional (correlation)	*HABDS or HERS*: peak of hip internal rotation (n.s.) peak of hip adduction (n.s.) peak of contralateral pelvic drop (n.s.) excursion of hip internal rotation (n.s.) excursion of hip adduction (n.s.) excursion of contralateral pelvic drop (n.s.)

*ANOVA* analysis of variance, *ES* effect size, *HABDS* hip abductor strength, *HERS* hip external rotator strength, *IQR* interquartile range, *f* female, *m* male, *N/A* not available, *n.s.* non-significant, r correlation coefficient, *RoM* range of motion, *VO2max* maximal oxygen consumption, *vs* versus, * statistically significant

### Experimental Characteristics

#### Hip Abductor and Hip External Rotator Strength (Independent Variable)

All studies measured HABDS. Eight of the 19 studies additionally measured HERS [19–21, 44, 46, 51, 53, 55].

##### Position

Thirteen studies measured HABDS in a side-lying position [20, 21, 40–46, 48, 50, 51, 53, 54], three in a standing position [19, 49, 52], and one in a supine position [55]. Another study did not report the testing position [47]. HERS was either measured seated [19, 20, 44, 51, 53, 55] or prone [21, 46, 55].

##### Measurement Device

Eleven studies recorded strength with a hand-held dynamometer [20, 21, 41, 43, 44, 46, 48, 50, 51, 53, 55] and eight with a stationary dynamometer [19, 40, 42, 45, 47, 49, 52, 54]. Most authors fastened their hand-held dynamometer with a non-elastic strap or anchoring station. Two used their devices manually [41, 55].

##### Contraction Type

Fourteen studies measured strength isometrically [19–21, 40, 42–44, 46, 48, 50, 51, 53–55], three concentrically [45, 49, 52], and two eccentrically [41, 47].

##### Force/Torque

While the output of the isokinetic and stationary dynamometers was torque, the hand-held dynamometers recorded a measure of force. Thus, most authors multiplied their force values by the length of the moment arm. Only a few authors used force directly as a predictor variable [21, 43, 44, 53]. All studies but one [21] normalized their strength values to body mass and two also to body height [41, 42].

##### Bilateral or Unilateral Measurements

Some studies measured strength only on the right side [20, 21, 41, 48, 53]. Others measured either right or left depending on which side was considered dominant [43, 54] or based on randomization [51]. However, most studies measured both sides and analyzed them either separately [44, 45, 50, 55] or combined [19, 47, 49]. The remaining studies did not report the testing side [40, 42, 46, 52].

#### Running Biomechanics (Dependent Variables)

All studies used reflective marker-based 3D motion capture to estimate running kinematic variables. Four of them combined kinematics with force plates to investigate running kinetics using external forces directly [20] or estimating internal joint moments through inverse dynamics based on external forces [44, 48, 53]. Running pace during testing was prescribed [20, 21, 40, 41, 46–48, 52–54] or self-selected [19, 42, 43, 49–52]. One study did both [52] and another based speed on ± 5% of the mean training pace [55]. Two studies did not report pace [45, 55] and oe study described only a range [44]. To compare paces between self-selected and prescribed, we calculated weighted means by dividing the sum of the products of pace and sample size by the total sample size. The weighted means were 3.18 m/s (*n* = 413) and 3.19 m/s (*n* = 213) for prescribed and self-selected paces, respectively. Moreover, participants ran either on the treadmill [20, 21, 40, 42, 43, 46, 47, 50–53] or over ground [19, 41, 48, 54] with a weighted mean pace of 2.99 m/s (*n* = 459) and 3.72 m/s (*n* = 167), respectively.

#### Study Designs and Statistics

There were two intervention studies addressing HABDS and HERS [44, 46]. One was a block-randomized controlled trial matching participants on age and running volume [46], while the other used no control group [44].

The remaining studies were all cross-sectional. Two of them included group comparisons [41, 43]. The first compared the eccentric HABDS of a group with small peak hip adduction angles to a group with large peak hip adduction angles [41]. The second compared the running biomechanics of those with the weakest to those with the strongest HABDS [43]. Another two studies performed multiple linear regressions [47, 54]. The remaining 13 studies calculated correlations between HABDS or HERS and different biomechanical variables.

#### Intervention Studies

The two intervention studies’ training protocols can be found in Table 2 [44, 46].

**Table 2:** Comparison of the two intervention programs

	Snyder et al. 2009 [44]	Willy and Davis 2011 [46]
Frequency	3 times/week	3 times/week
Duration	6 weeks	6 weeks
Aim	Increasing HABDS and HERS	Increasing HABDS and HERS; improving joint alignment
Trained sides	Right	Right and left
Hip exercises	standing on the right leg moving left hip towards and away from pelvic drop, external rotation, or internal rotation	Week 1–2: non-weight-bearing (side-lying hip external rotation with hip extension, hip abduction straight leg raises, and resistance band clamshells); week 3–6: weight-bearing (contralateral pelvic hike, sidestepping with a resistance band, and standing hip abduction with hip external rotation and pelvic hike)
Training device(s)	Strap around the waist connected to a cable column	Resistance bands or weight bearing
Progression	Increasing weights of cable column	Increasing resistance of the bands and increasing level of difficulty
Contraction type	Concentric and eccentric	Concentric, eccentric, and isometric
Intensity	N/A	Adjusted to cause fatigue after two sets
Other exercises	None	Squats and movement education program
Strength changes	HABDS by 12.8% (ES = 0.68; *p* = 0.01) HERS by 23.1% (ES = 1.0; *p* < 0.000)	HABDS by 41.7% (ES = 1.39; *p* = 0.006) HERS by 24.1% (ES = 0.62; *p* = 0.003)
Control group	No	Yes

*ES* effect size, *HABDS* hip abductor strength, *HERS* hip external rotator strength, *N/A* not available

Both intervention groups trained three times per week for six weeks and achieved significant gains in HABDS and HERS. However, their programs differed substantially. Snyder et al. (2009) trained the right side [44], whereas Willy and Davis (2011) implemented a bilateral program that also incorporated squat exercises and a movement education component, using mirror feedback and verbal cues to improve joint alignment [46]. Notably, only one of the two studies used a control group design [46].

## Discussion

### Summary

This scoping review identified and mapped 19 studies examining the influence of HABDS and HERS on running biomechanics in healthy runners. Taken together, the available evidence does not provide strong or consistent support for a meaningful relationship between HABDS or HERS and running biomechanics in healthy runners. Although several plausible biomechanical mechanisms have been proposed, findings across joints and planes are heterogeneous, frequently based on single studies, and often dependent on specific subgroups or experimental conditions. Where associations were observed, they were not consistently replicated and were rarely supported by controlled intervention evidence. Importantly, the lack of consistent findings should not be interpreted as inconclusive reporting, but rather as evidence that current data do not support a clear or robust link between HABDS or HERS and running biomechanics. Thus, the prevailing assumption that hip strengthening alone leads to systematic biomechanical changes during running is not substantiated by the existing literature. The studies assessed a wide variety of biomechanical variables, drawn from an equally diverse set of gait cycle characteristics. Such variability makes direct comparisons difficult and limits the number of studies reporting the same parameter in a consistent manner. The hip, knee, and pelvis received the greatest attention, whereas the trunk, tibia, and ankle were investigated less frequently. Commonly reported gait cycle characteristics included peak angles, range of motion, angular excursion, and peak velocity, while only a few studies analyzed joint moments. The studies differed considerably in their experimental designs and methodological approaches, making it even more challenging for evidence synthesis.

### Outcome Results

#### Trunk

Several authors have proposed that runners with weak HABDS may compensate by leaning the trunk toward the stance limb, thereby reducing hip abductor demand [44, 47, 51]. While this mechanism is theoretically plausible and often cited, current evidence does not support this link [19, 52]. Instead, isolated findings suggest possible links between HABDS and trunk rotation [52] and trunk flexion in females [19]. These single-study observations highlight intriguing directions, but replication is lacking, and the overall role of trunk movements as compensatory strategies remains uncertain. More targeted investigations are needed to clarify whether trunk adaptations mitigate or conceal the influence of hip strength on running mechanics and whether these relationships differ between sexes.

#### Pelvis

A commonly proposed theory is that weak hip abductors and external rotators contribute to pelvic instability, particularly pelvic drop, which in turn may trigger a cascade of compensatory movements from proximal to distal segments [14, 16]. However, many studies have not confirmed this association. The few that did, however, support this theory as they link greater HABDS to greater pelvic stability (i.e. less movement), not only in the frontal but in all three planes [19, 52].

Because cross-sectional evidence remains equivocal, more intervention studies are needed to establish a causal link between HABDS or HERS and pelvic mechanics. To date, only one such study has been conducted [46], but its findings may have been confounded by the inclusion of a concurrent movement education program, which could have interfered with strength progression. Focusing on strengthening alone may not only yield greater strength gains but also allow researchers to attribute biomechanical changes directly to improvements in muscle strength.

#### Hip

Hip movements received the greatest attention among the included studies, especially hip abduction/adduction. From a biomechanical perspective, weak hip abductors are thought to contribute to excessive hip adduction [14]. Yet, despite extensive investigation, the evidence supporting this idea remains limited. The few cross-sectional studies in favor of the theory linked greater HABDS with reduced hip adduction movement and velocity [19, 48, 49]. This suggests that increasing HABDS could improve control of frontal plane hip motion during running. Surprisingly, however, one of the two intervention studies testing this assumption reported the opposite with an increase of hip adduction range of motion after strengthening [44]. But with no control group, it is unclear whether this change was caused by the intervention or by other factors.

Sex may add another layer of complexity. While one association was only observed in females within a mixed-sex sample [19], the other two reported associations were derived from male only samples [48, 49]. Again, to gain a more nuanced understanding, future research should consistently include both sexes and analyze them separately.

#### Knee

The knee was the second most frequently studied joint after the hip. This is not surprising, since the majority of running-related injuries occur at the knee [5]. One theory is, that weak hip abductors may increase either varus or valgus moment, depending on whether pelvic drop is compensated by leaning the trunk towards the stance limb [14]. These altered forces could place additional stress on the knee joint, potentially leading to overuse running injuries [17].

What remains uncertain, however, is whether HABDS or HERS directly influence knee mechanics during running. Both have been related to injury risk, but current evidence suggests that a direct link between HABDS and knee mechanics is only present under specific conditions, such as fatigue [48], limb asymmetry [45], or being in a particular subgroup of runners [47].

Whether HABDS or HERS training can improve knee joint loading remains unclear. It appears promising that the knee abduction moment could be reduced after a hip abduction and hip external rotation strengthening program [44], but without a control group it is not possible to attribute those changes to the intervention itself or other factors. To clarify this relationship, future research should investigate HABDS and HERS under well-defined conditions and ensure the inclusion of control groups in training studies.

#### Tibia

Only one study examined lower leg movements with no significant correlation between HABDS and tibial inclination at foot strike [45]. The authors did not provide a rationale for exploring this association, suggesting it may have been an exploratory sub-analysis. This leaves open the broader question of whether HABDS or HERS meaningfully influences tibial mechanics.

A commonly proposed idea is that gluteal muscle weakness could contribute to altered running kinematics, including external tibial rotation [16]. However, none of the included studies directly assessed tibial rotation. This omission may reflect the common methodological limitation that this variable is highly sensitive to error when estimated with skin-marker models [57]. Given its potential relevance for understanding injury mechanisms, tibial movements, specifically tibial rotation, may be considered in future studies. However, their interpretation must account for measurement error [57].

#### Ankle

Only two studies investigated ankle movements, but both reported promising results [44, 45]. Collectively, their findings suggest that proximal muscle strength may contribute to greater ankle stability and reduced joint stress. Nevertheless, caution is warranted. Correlation does not imply causation, and the available intervention study lacked a control group, limiting the strength of its conclusions. Given that the ankle represents the third most common site of running-related injury [5] and the few preliminary studies reporting promising findings, there is a need for further research to clarify the link between HABDS or HERS and ankle mechanics.

### General Characteristics

Running level may influence how HABDS and HERS relate to running biomechanics. Fifteen studies included recreational runners, but interestingly, the remaining four studies on cross-country runners reported significant correlations with either HABDS [45, 49, 52, 55] or HERS [55]. Among recreational runners, the study with the highest training volume also observed significant associations [19]. However, positive findings were not limited to high-mileage athletes [43, 44]. This pattern suggests that running level is unlikely to be the sole determinant, but it may shape the strength or consistency of observed associations.

### Experimental Characteristics

#### Hip Abductor and Hip External Rotator Strength (Independent Variable)

HABDS and HERS may have an independent effect on running biomechanics, since studies that analyzed both found significant associations either for one or the other [19, 55]. Therefore, we suggest that researchers include both variables in future investigations. The gained knowledge from a separate analysis may help practitioners to design more efficient training programs that specifically target the muscles or movements that actually need strengthening.

#### Position

The position used to measure strength may influence the observed association between HABDS or HERS and running biomechanics. One could argue that testing should replicate the functional demands of running. Supporting this idea, all studies that assessed HABDS in a standing position reported significant associations [19, 49, 52]. In contrast, only three of 14 studies that used a side-lying position found significant effects [43, 44, 48]. This suggests that while testing position may not be the decisive factor, it likely contributes to whether significant associations are detected. HERS was measured seated or prone without any indication of affecting the findings.

#### Measurement Device

There is no clear indication that the choice of measuring device had an influence on the outcome. Although four studies that used an isokinetic dynamometer reported significant results [45, 47, 49, 52], hand-held dynamometry can be considered a reliable and valid instrument for measuring muscle strength, even in comparison to isokinetic dynamometry, which is the gold standard [58].

#### Contraction Type

The idea that eccentric rather than isometric HABDS might reflect hip abduction demands during running better seems plausible [41]. However, the findings of the two studies measuring eccentric instead of isometric strength did not show a stronger relationship between eccentric HABDS and running biomechanics [41, 47] compared to the other studies that measured isometric or concentric HABDS.

#### Force/Torque

With only a few exceptions [21, 43, 44, 53], there was a consensus among studies to consider the moment arm. Similarly, most studies normalized strength values to body mass or height, with only one exception doing neither [21]. This allows a better comparison between studies and reduces bias from anthropometric parameters. Researchers conducting future studies should be aware of these methodological considerations.

#### Bilateral or Unilateral Measurements

Based on the current findings, it is difficult to judge the effect of the strength testing side. Only one of the four studies that measured both sides to analyze them separately [44, 45, 50, 55] reported different associations depending on the tested side [45].

### Running Biomechanics (Dependent Variable)

All studies used 3D motion capture to describe running kinematics. Interestingly, one of the three studies that additionally performed inverse dynamics found significant changes in joint loading for a movement where the kinematic variable was unaltered [44]. This might encourage future researchers to use inverse dynamics in addition to motion capture for a deeper insight into possible associations between hip strength and internal joint loading that may relate to injury risk.

Running pace had no noticeable impact on study outcomes. In an exploratory sub-analysis (not shown in the Results section), we ranked the studies by running pace, but that did not reveal any connection between pace and results either. Ultimately, the decisive factor may not be load but strain. If the hip abductors are influencing running biomechanics, they might only do so under sufficient strain. This could be accomplished by either increasing the load with a higher pace or by reducing load tolerance through fatigue. One study tested the latter by introducing fatigue with a 15% reduction in HABDS torque after a graded exercise test and subsequent running to exhaustion [48]. This concept revealed associations between HABDS and certain biomechanical variables only in the presence of fatigue, but not in the rested state [48]. A more radical approach to reducing HABDS could be to perform a superior gluteal nerve block injection [59]. However, even a 26% HABDS redction did not change biomechanics during walking [59]. It is possible that the remaining 74% of HABDS werestill sufficient to enable biomechanically unaltered walking. Such a reduction in strength might only affect locomotion at higher speeds. If that holds true, it might be possible to strain the hip abductors enough by just running faster. Both approaches, running under fatigue or at higher paces, need further investigation to understand the true connection between HABDS and running biomechanics.

### Recommendations for Future Research

#### Sex-Specific Effects

While females have a higher overall incidence of running-related injuries compared to males [5], they also demonstrate distinct biomechanical patterns [60]. Specifically, female runners tend to exhibit less knee flexion, but greater hip flexion, hip adduction, and hip internal rotation [60]. If biomechanics contribute to injury mechanisms, and if hip strength influences biomechanics [17], it is reasonable to explore sex-specific associations between HABDS or HERS and running biomechanics. Among the studies included, seven used mixed-sex samples [19, 50–55] and one of them reported associations that were sex-specific [19]. Yet, the current evidence base remains limited and more research is needed to clarify how sex, strength, and biomechanics interact in the context of running.

#### Statistical Correction

Since the majority of studies conducted Pearson correlations between HABDS or HERS and multiple biomechanical variables, this might have led to a Type I error inflation if no statistical correction was applied [61]. We searched for reports of such corrections and among the six correlation studies [19, 45, 48, 49, 52, 55] that found significant correlations, only one explicitly mentioned statistical correction [19]. It is beyond the scope of this review to conduct a comprehensive quality appraisal. However, future studies should consider statistical correction when they perform multiple statistical tests.

#### Loss of Information

A limitation of the included studies might be the loss of information by only analyzing single gait cycle characteristics, such as peak angle [54] or range of motion [49]. It remains unclear if they are representative of the investigated phenomenon. For example, no association between HABDS and knee abduction/adduction range of motion [49] does not exclude that there could still be an association between HABDS and any other characteristic of the knee abduction/adduction curve. A solution to that problem, at least for group- or pre- to post-comparisons, could be using time-series analysis like statistical parametric mapping [62] that considers the entire stance phase or even the whole gait cycle.

#### Assumption of Linearity

Another limitation of the included studies was the assumption of linearity. Pearson correlation, multiple linear regression, and ANOVA assume a linear relationship between independent and dependent variables. Whether this reflects the true relationship between HABDS or HERS and running biomechanics is unclear. Assuming there is such a relationship, we suggest that there might be a lower and an upper threshold for the required strength. Since HABDS or HERS are not performance-limiting factors, they must only be strong enough to maintain a normal running pattern [40]. According to that theory, only strength below a particular threshold might be a limitation, and strength beyond the requirements would only contribute under extreme circumstances, such as at higher running paces or under fatigue. Evidence from one study supports the idea of a lower threshold, where only the weakest individuals showed 4° greater knee abduction compared to the strongest [43]. The theory that the upper threshold can shift under certain conditions is supported by another study, where significant associations only revealed themselves with fatigue [48]. This implies that strength was sufficient in the rested state but insufficient after an exhausting run. Future researchers may consider this in their study designs and statistics.

#### Construct Validity

Another issue might be the construct validity and the question of whether maximum HABDS or HERS are representative of the repetitive strength demands during running. Several authors suggested that strength endurance might better predict running biomechanics [40, 50]. Associations might further remain undetected because common strength testing modes do not represent the ballistic demands during the stance phase of running [41]. However, we could not identify any obvious relationship between outcomes and the different strength testing modes. The assumption that eccentric strength would show a stronger relationship with running biomechanics than isometric strength was not supported [41, 47].

#### Compensatory Movements

As discussed earlier, the influence of hip strength on lower body kinematics could have been obscured by a compensation mechanism where hip abduction demands are reduced by leaning the trunk towards the stance limb [44, 47, 51]. However, all but two studies [19, 52] omitted to monitor lateral trunk movements. A similar compensation could happen when other muscles crossing the hip compensate for hip abductor or hip external rotator weakness [40]. For this reason, several studies have recommended using electromyography to investigate whether weak hip abductors lead to altered activation patterns [19, 40, 50].

### Limitations of the Scoping Review

The choice to conduct a scoping review prioritized breadth over depth. By framing a broad research question, we could not provide detailed evaluations of individual biomechanical variables. Instead, this review offers a comprehensive overview of the field and highlights where the evidence base is strongest, pointing future researchers toward the most promising areas for more focused, in-depth analysis.

At least three studies described the recording of variables in their methods but failed to report them in their results [20, 51, 53]. As a result, our review may have missed relevant evidence from studies that did not fully report all measured outcomes. This may reflect variables that were measured but not analyzed, or variables that were both measured and analyzed but not reported.

## Conclusions

In conclusion, this scoping review found insufficient evidence to support a consistent association between HABDS or HERS and running biomechanics in healthy individuals. Likewise, there is no convincing evidence that increasing these strength capacities alone results in systematic or predictable changes in running kinematics or kinetics. While isolated findings suggest potential relationships under specific conditions, the overall body of evidence does not support commonly held assumptions regarding the biomechanical effects of hip strengthening. These findings highlight the need to reconsider the expectation that hip abductor or hip external rotator strengthening interventions alone can modify running biomechanics and emphasize the importance of well-controlled intervention studies to clarify under which conditions, if any, such effects may occur.

## Data Availability

Not applicable.

## Abbreviations

HABDS: Hip abductor strength
HERS: Hip external rotator strength
